# Left Atrial Appendage Occlusion: A Therapeutic Option in a Patient With Atrial Fibrillation and Hereditary Hemorrhagic Telangiectasia

**DOI:** 10.7759/cureus.15367

**Published:** 2021-06-01

**Authors:** Luciana Frade, Cláudio Gouveia, Renato Guerreiro, Susana Jesus, Candida Fonseca

**Affiliations:** 1 Internal Medicine, Hospital de São Francisco Xavier, Lisboa, PRT; 2 Internal Medicine, Centro Hospitalar de Lisboa Ocidental, Lisbon, PRT

**Keywords:** hereditary hemorrhagic telangiectasia, rendu-osler-weber syndrome, atrial fibrillation, left atrial appendage occlusion, bleeding disorders

## Abstract

Hereditary hemorrhagic telangiectasia (HHT) is a predominantly inherited disorder of blood vessel structure, characterized by mucocutaneous telangiectasias, multiple arteriovenous malformations, and frequent epistaxis. A 67-year-old female with atrial fibrillation and high thromboembolic risk (CHADs2Vasc2: 4) with renal arterial thrombosis started oral anticoagulation (OAC). The patient had multiple episodes of heavy nasal and gastrointestinal bleeding (requiring multiple blood transfusions) such that OAC had to be interrupted, and a complementary investigation led to the diagnosis of HHT. Due to concomitant high thromboembolic and hemorrhagic risks, the patient was proposed left atrial appendage occlusion as an alternative to OAC intolerance. After the procedure, there were no new episodes of bleeding or thrombotic events.

## Introduction

Hereditary hemorrhagic telangiectasia (HHT), also known as Rendu-Osler-Weber syndrome, is a rare autosomal dominant disorder first described in 1896 [1], characterized by mucosal, visceral, and dermal telangiectasias as well as visceral arteriovenous malformations (AVMs) of various organs. A recent study [2] revealed that HHT patients are more prone to develop atrial fibrillation (AF), most probably due to frequent systemic AVMs, iron deficiency anemia, as well as hypoxemia resulting in disorderly circulation. Such patients are recommended to be on chronic oral anticoagulation (OAC) to prevent thromboembolic events. However, due to a high risk of both internal and external bleeding, OAC can be difficult to manage and is not the ideal treatment. Left atrial appendage occlusion, a minimally invasive percutaneous procedure, constitutes a legitimate alternative treatment when AF is present.

## Case presentation

In June 2016, a 67-year-old caucasian female, with a clinical history of hypertension, iron deficiency anemia, duodenal angiectasia, and occasional nasal bleeding, came to our Emergency Department (ED) complaining of palpitations. No other complaints were reported by the patient. On physical examination, the patient had a dysrhythmic pulse, tachycardic heart sounds, blood pressure of 146/100 mmHg, and heart rate of 150 beats/minute (irregularly irregular). Blood tests were collected at the ED, revealing hemoglobin (Hb) of 12.1 g/dL, creatinine 0.82 mg/dL, N-terminal pro-brain natriuretic peptide 3,032 pg/mL, and troponin-I 29-39 μg/mL. Arterial blood gas analysis showed hypoxemia with no other relevant abnormalities. Chest X-ray revealed upper zone vessel prominence and pulmonary interstitial edema, electrocardiogram (EKG) showed AF at 110 beats/minute, and chest computed tomography angiography excluded pulmonary thromboembolism.

The patient was then admitted to our Internal Medicine ward with a diagnosis of heart failure decompensated by AF (CHADs2Vasc2: 4; HAS-BLED: 3) and anticoagulation was initiated. Transthoracic echocardiography revealed preserved left ventricle ejection fraction (55%), left atrial dilation, and moderate aortic valve stenosis (mean gradient: 35 mm). During the hospital stay, the patient suffered acute left renal artery thrombosis and developed acute renal failure. Acetylsalicylic acid (ASA) 150 mg was prescribed to complement the treatment with low-molecular-weight heparin (LMWH) already prescribed in the ED (dose adjusted to renal clearance). The patient developed severe episodes of epistaxis, needing multiple cauterization procedures; ASA was removed from the treatment regimen. She was prescribed warfarin and discharged under LMWH for warfarin bridging.

One month later, the patient was again hospitalized due to melena and tiredness. On admission, she presented with Hb of 5.9 g/dL and international normalized ratio (INR) of 3.1. OAC was suspended, and vitamin K was administered. The patient underwent three blood transfusions. Endoscopic examination revealed bleeding duodenum and jejunum angiectasias that were treated with argon plasma coagulation (APC). Considering the high thrombotic risk, she restarted OAC.

Four months later, she was readmitted due to severe epistaxis and melena. Her blood tests revealed Hb of 7.3 g/dL, iron 24 μg/dL, ferritin 86 ng/mL, transferrin saturation 8%, and INR 3.4. Anticoagulation was suspended and she was given two blood transfusions and 1,500 mg of intravenous ferric carboxymaltose. Endoscopic examination showed new bleeding angiectasias, and again she was treated with APC. As soon as LMWH was reintroduced, epistaxis recurred; hence, new local cauterization was done and anticoagulation was stopped.

**Figure 1:**
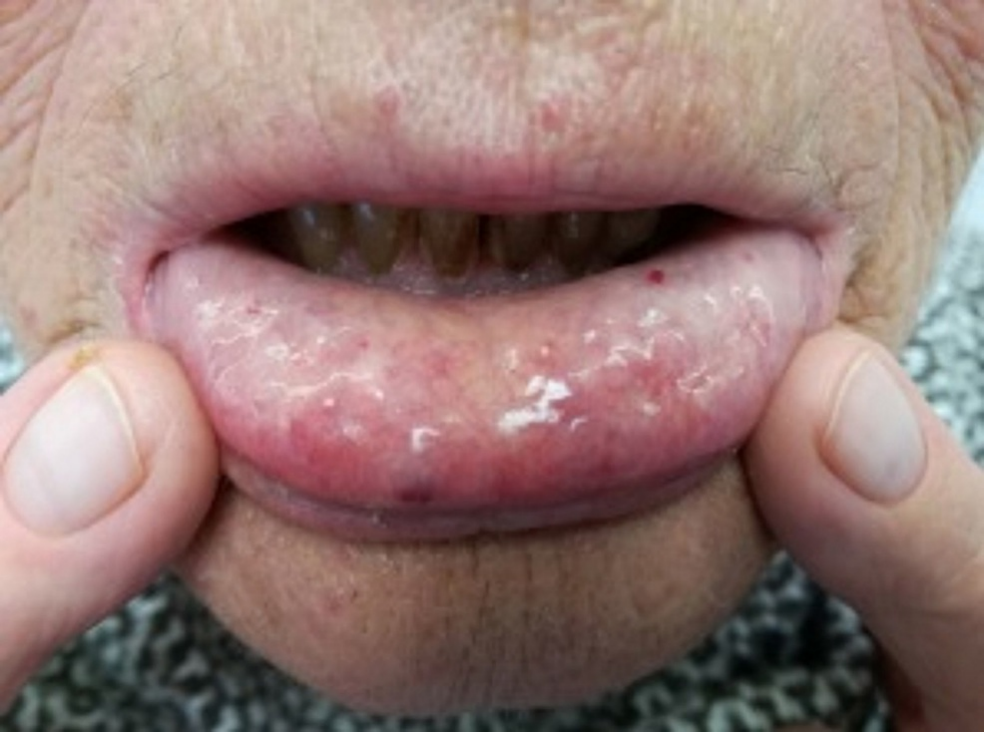
Mucosal telangiectasis (lower lip).

**Figure 2:**
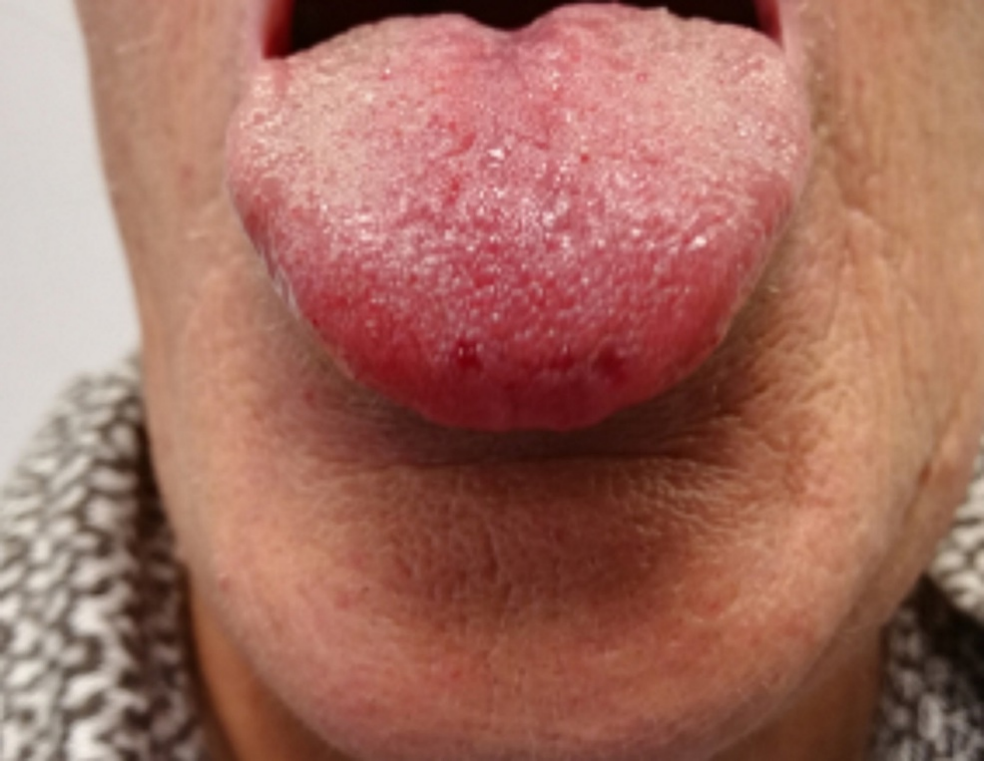
Tongue telangiectasis.

**Figure 3:**
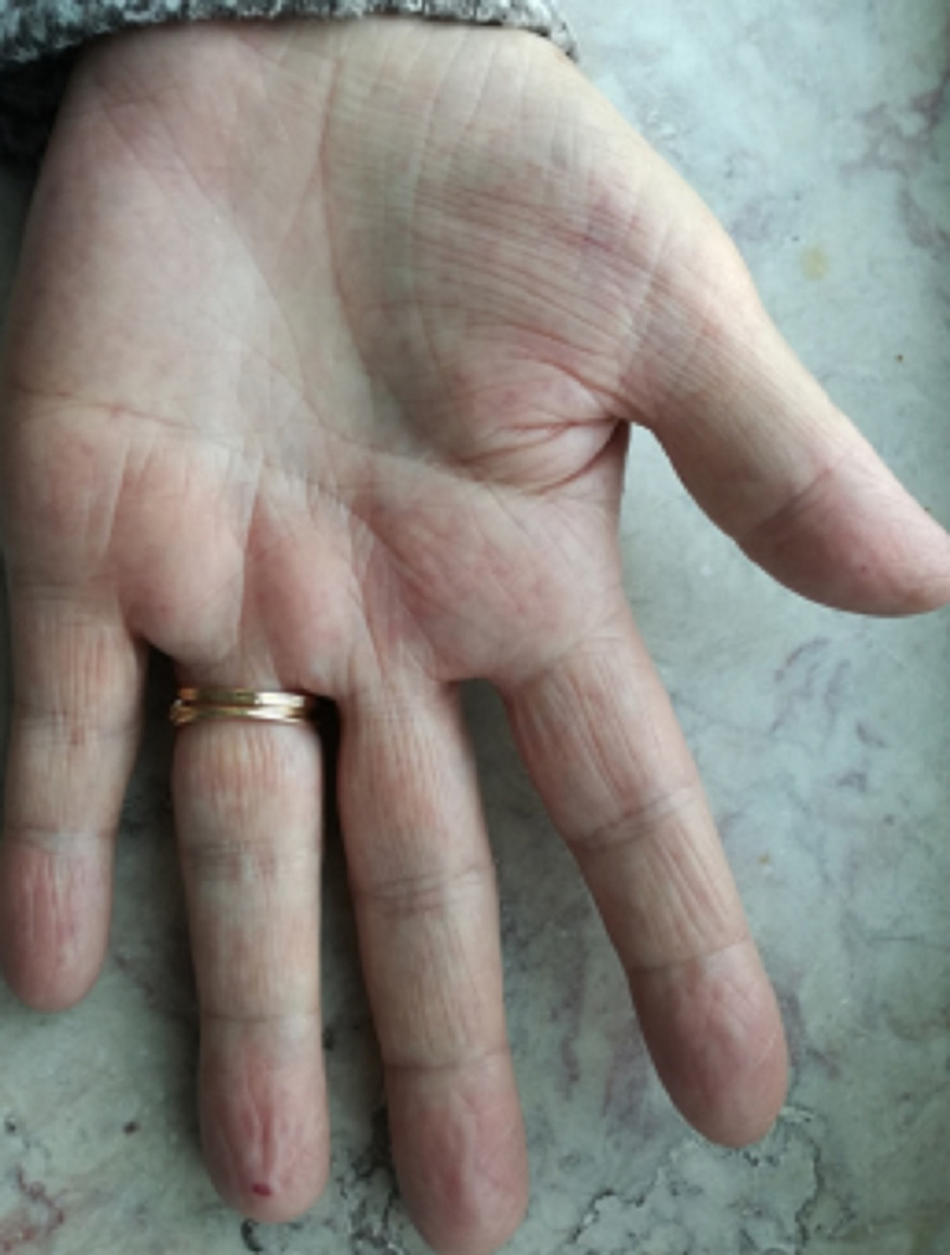
Palmar telangiectasis.

**Figure 4:**
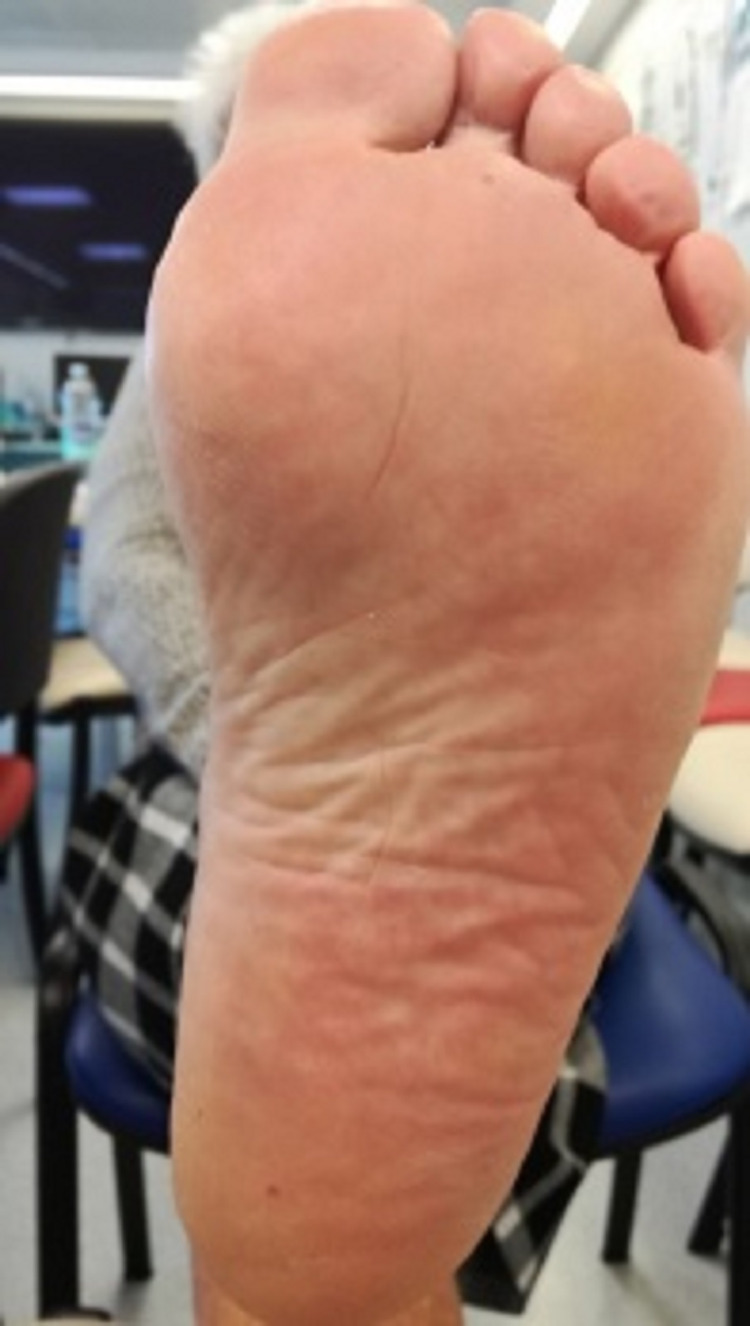
Plantar telangiectasis.

Altogether, the recurrent epistaxis, multiple visceral angiectasias, careful physical examination revealing lip (Figure 1), tongue (Figure 2), palmar (Figure 3), and plantar telangiectasias (Figure 4), as well as family history of nasal bleeding (father and sister with frequent epistaxis), made the authors arrive at the conclusion that the patient met the criteria for HHT.

Due to high thrombotic risk and OAC intolerance, the patient’s case was discussed with interventional cardiology and left atrial appendage occlusion (LAAO) was proposed. After transesophageal echocardiography to determine device sizing and exclude thrombus, LAAO was successfully performed and the patient was discharged on ASA 100 mg/id and clopidogrel 75 mg/id for six weeks after the procedure. One month later, the patient again complained of melena and tiredness. Her Hb was 6.1 g/dL, and she received four blood transfusions and 1,000 mg intravenous ferric carboxymaltose. Complete gastrointestinal endoscopic studies revealed no bleeding lesions. After discussing the patient with the cardiologist, ASA was suspended, and clopidogrel was maintained for six weeks after the procedure. After the six-week period, the patient stopped the antiplatelet agent. Currently, the patient’s EKG shows sinus rhythm and the patient has not had any new major bleeding or thrombotic episodes. Her hemoglobin is stable at 12 g/dL.

## Discussion

The authors report the case of a 67-year-old woman with concomitant high thromboembolic and hemorrhagic risk, due to AF and HHT, presenting with an acute thromboembolic requiring OAC subsequent serious recurrent blood loss (leading to iron deficiency anemia demanding multiple blood transfusions). HHT diagnosis was established according to the clinical criteria. The diagnosis was established based on recurrent epistaxis, gastrointestinal bleeding, and mucocutaneous involvement. Moreover, in our patient, three out of four of the Curaçao Criteria, launched in 1999, were present. These include frequent epistaxis, multiple telangiectasias at typical sites (lips, oral cavity, fingers, nose), visceral lesions (gastrointestinal tract, pulmonary, cerebral, and spinal), and family history of a first-degree relative with HHT. Most commonly, the patient seeks a doctor due to severe bleeding and its associated symptoms (e.g., extreme tiredness), and there is usually a need for innumerable blood transfusions. Because of this clinical presentation and sometimes life-threatening bleeding predisposition, arbitration on starting OAC in patients with HHT and high-risk AF should be based on a careful patient-directed risk assessment of both thromboembolic and hemorrhagic complications [3]. This diagnosis gave the patient a high hemorrhagic risk; however, there was also an unequivocal indication for anticoagulation to treat a serious acute thromboembolic event, left renal artery thrombosis and further recurrence.

Maintaining or discontinuing OAC was a clinical challenge. As recommended by guidelines, the benefit of OAC was determined by the risk of hemorrhage over the risk of thromboembolic events. It was discussed with the patient, and after multiple serious bleeding episodes, OAC was suspended, as it was no longer an option, and the patient was proposed LAAO. According to the European Society of Cardiology Guidelines, LAAO may be considered in patients with AF and contraindications for long-term OAC therapy such as thrombocytopenia or known coagulation defect, recurrent bleeding, prior severe bleeding (including intracranial hemorrhage), poor compliance or intolerance with anticoagulation therapy, and high risk of falls or prior falls resulting in injury [3].

After LAAO, dual antiplatelet therapy is fundamental in the first months when endothelialization of the device is not yet complete, followed by indefinite treatment with ASA 100 mg id [3]. As the patient could not tolerate OAC, it was decided, after discussion with the Cardiology Department, to treat the patient only with dual antiplatelet therapy as HHT patients tolerate antiplatelet therapy better than OAC [4]. Antithrombotic therapy after LAAO should also be individualized considering the patient’s history of bleeding, comorbid medical conditions, the completeness of LAAO, and potential for device thrombosis [5,6]. On dual antiplatelet therapy, there were still episodes of gastrointestinal blood loss and anemia exacerbation, and ASA had to be suspended after four weeks. The patient completed the six-week antiplatelet scheme with just clopidogrel. To date, the patient has not had any new thromboembolic or hemorrhagic events and is maintaining stable hemoglobin levels.

## Conclusions

This case report demonstrated the safety and efficacy of the left atrial appendage closure as an alternative strategy in patients with an indication for OAC who are not able to tolerate them due to bleeding episodes, as it occurs in patients with HHT.

At present, LAAO in HHT patients remains anecdotal; only a few patients undergo the procedure, and larger numbers are needed to reveal the risks and benefits of this therapeutic approach. However, it is well known that thromboembolic complications in patients with AF arise most commonly from the left atrial appendage as percutaneous transcatheter LAA occlusion is an attractive alternative when OAC is not possible.
